# Reply to B Stansfield

**DOI:** 10.1016/j.advnut.2025.100436

**Published:** 2025-05-22

**Authors:** Alessandra Consales, Carlo Agostoni, Roberta Cazzola, Roberta Ottria, Maria Lorella Giannì

**Affiliations:** 1 Department of Clinical Sciences and Community Health, Dipartimento di Eccellenza 2023-2027, University of Milan, Milan, Italy; 2 Pediatric Unit, Fondazione IRCCS Ca' Granda, Ospedale Maggiore Policlinico, Milan, Italy; 3 Department of Biomedical and Clinical Sciences, University of Milan, Milan, Italy; 4 NICU, Fondazione IRCCS Ca’ Granda Ospedale Maggiore Policlinico, Milan, Italy

Dear Editor:

We would like to thank Professor Stansfield for his interest in our recent review [1]. We greatly appreciate his acknowledgment of our work and commend the contribution his research has made to the growing understanding of zinc concentrations in human milk.

We regret that Professor Stansfield’s relevant study [2] was not cited in our review. Although every effort was made to be comprehensive, narrative reviews are not exhaustive by design, thus some valuable contributions may inadvertently be missed. We are thankful to Professor Stansfield for sharing supplementary analyses of his data, which enrich our collective understanding, and we are pleased that his results support the conclusions presented in our review.

Professor Stansfield’s findings show that the stage of lactation, rather than gestational age alone, is a critical variable to account for when measuring zinc concentrations in human milk. Failing to consider it may lead to inaccurate conclusions, especially when comparing term and preterm human milk samples. For instance, comparing transitional milk from a mother who delivered at term with mature milk from a mother who delivered prematurely may misattribute any zinc concentration differences to gestational age at birth, when in fact they could just as well arise from the distinct stages of milk’s maturation.

It has been previously shown – and Professor Stansfield’s data support – that zinc concentrations decrease rapidly in the first month of lactation, both in term and preterm milk, eventually reaching a plateau [3]. A steeper decline in zinc concentrations with decreasing gestational age at birth may explain the lack of difference at later postnatal age observed by Professor Stansfield between samples from mothers who delivered extremely and very preterm infants.

The temporal changes observed in human milk zinc concentrations are not merely biochemical curiosities; they have important clinical implications, as they may guide appropriate fortification strategies or supplementation practices in preterm infants. This consideration becomes even more critical when preterm infants are fed donor human milk, which typically results from pasteurized pooled samples collected during late lactation from mothers who delivered at term. Furthermore, when interpreting human milk micronutrient adequacy for preterm infants, the different daily volumes consumed by preterm and term infants – which ultimately determine actual zinc intake – should be considered as well.

The inconsistencies found across studies comparing zinc concentrations in term and preterm milk underscore the importance of designing appropriate studies that control for both gestational and postnatal age (i.e., lactation stage), as well as other potential confounders, and reinforce our previous conclusion that further well-conducted research is essential to clarify this aspect [1].

Interestingly, this debate recalls a similar discussion years ago regarding long-chain polyunsaturated fatty acid content in human milk. Then, too, although some researchers described greater long-chain polyunsaturated fatty acid content in preterm compared with term milk, others suggested that the fatty acid composition of human milk might be influenced by factors other than gestational age alone, such as lactation stage, maternal diet and stores, and milk volume supplied to the infant, as previously reviewed [4,5]. This parallel reminds us that careful consideration of human milk dynamic and context-dependent nature is critical for accurately interpreting data on its nutrient composition.

## Funding

This study was partially funded by the Italian Ministry of Health, Current research IRCCS.

## Conflict of interest

The authors report no conflicts of interest.
